# Enhancing Nurse–Robot Engagement: Two-Wave Survey Study

**DOI:** 10.2196/37731

**Published:** 2023-01-09

**Authors:** Gen-Yih Liao, Tzu-Ling Huang, May-Kuen Wong, Yea-Ing Lotus Shyu, Lun-Hui Ho, Chi Wang, T C E Cheng, Ching-I Teng

**Affiliations:** 1 Department of Information Management Chang Gung University Taoyuan Taiwan; 2 Department of Nursing Chang Gung Memorial Hospital, Taoyuan Branch Taoyuan Taiwan; 3 Department of Information Management National Central University Taoyuan Taiwan; 4 Department of Physical Medicine and Rehabilitation Chang Gung Memorial Hospital, Linkou Taoyuan Taiwan; 5 School of Nursing, College of Medicine Chang Gung University Taoyuan Taiwan; 6 Department of Nursing Chang Gung Memorial Hospital, Linkou Taoyuan Taiwan; 7 Department of Nursing Chang Gung University of Science and Technology Taoyuan Taiwan; 8 Department of Logistics and Maritime Studies The Hong Kong Polytechnic University Kowloon Hong Kong; 9 Graduate Institute of Management Chang Gung University Taoyuan Taiwan; 10 Department of Business and Management Ming Chi University of Technology New Taipei City Taiwan

**Keywords:** healthcare, health care, eHealth, digital heath, health technology, personal innovativeness, robot, structural equation modeling, survey, intelligent technology, smart technology, robotics, nurse, nursing, attitude, engagement, healthcare professional, benefit, Taiwan, Asia

## Abstract

**Background:**

Robots are introduced into health care contexts to assist health care professionals. However, we do not know how the benefits and maintenance of robots influence nurse–robot engagement.

**Objective:**

This study aimed to examine how the benefits and maintenance of robots and nurses’ personal innovativeness impact nurses’ attitudes to robots and nurse–robot engagement.

**Methods:**

Our study adopted a 2-wave follow-up design. We surveyed 358 registered nurses in operating rooms in a large-scale medical center in Taiwan. The first-wave data were collected from October to November 2019. The second-wave data were collected from December 2019 to February 2020. In total, 344 nurses participated in the first wave. We used telephone to follow up with them and successfully followed-up with 331 nurses in the second wave.

**Results:**

Robot benefits are positively related to nurse–robot engagement (β=.13, *P*<.05), while robot maintenance requirements are negatively related to nurse–robot engagement (β=–.15, *P*<.05). Our structural model fit the data acceptably (comparative fit index=0.96, incremental fit index=0.96, nonnormed fit index=0.95, root mean square error of approximation=0.075).

**Conclusions:**

Our study is the first to examine how the benefits and maintenance requirements of assistive robots influence nurses’ engagement with them. We found that the impact of robot benefits on nurse–robot engagement outweighs that of robot maintenance requirements. Hence, robot makers should consider emphasizing design and communication of robot benefits in the health care context.

## Introduction

### Overview

Artificial intelligence–based technologies exhibit useful functions in medical contexts [1,2], and could share nursing workloads, thus helping reduce nurses’ time pressure, and alleviating the global issue of nurse shortages. For example, robots can be used to facilitate patient care delivery to mitigate shortages in the care workforce [3]. Past studies have indicated the important role of health care practitioners’ attitudes and perceptions on technology implementation [4] and nurses’ attitudes to health information technology [5]. That is, if nurses do not welcome the presence of the robot, they are unlikely to assist robots through maintenance, positioning, or ensuring their smooth operations. Hence, robots may hardly help health care practice. Moreover, previous research has shown that the majority of clinical professionals do not believe that artificial intelligence–based technologies can provide clinically relevant assistance [6]. This is a warning to both scholars and practitioners that clinical professionals may not engage (or may not sufficiently engage) with artificial intelligence–based technologies. However, the literature has not offered means to effectively increase clinical professionals’ engagement in the use of these technologies, indicating a research gap.

We attempted to address this gap by examining how features of artificial intelligence–based technologies could increase clinical professionals’ engagement with them. That is, the aim of this study is to examine how robot benefits, robot maintenance, and nurses’ personal innovativeness impact nurses’ attitudes toward robots and nurse–robot engagement. We have compiled the definitions of the study variables in Table 1.

**Table 1:** Definitions of the study concepts.

Concept	Definition
Robot benefits	How robots help nurses optimize their time and effort to implement nursing care
Robot maintenance	How robots consume nurses’ time and effort that could be used on other tasks
Personal innovativeness	The tendency to try new technologies
Attitudes toward robots	Overall evaluation of robots
Nurse–robot engagement	Intention to use robots and help ensure robots’ smooth operations

### Background

Artificial intelligence–based technologies have started to reshape how health care is delivered. Such technologies have frequently been implemented in the form of robots. Robots have been devised to assist various activities: social interaction (social robots), moving of objects (mobility robots), health education (digital health care assistant chatbots), or exercise sessions for older adults. Robots can also serve as a distraction tool for pain management and facilitate conversation and rapport [7], indicating that robots have the potential to enhance nursing practice. This potential points to the advantages of accepting robots into nursing practice.

The potential of improving clinical practice with robots does not guarantee their acceptance. Moreover, nurses have experienced stress and felt annoyed toward technology use in practice [5]. Hence, research is needed to understand how to increase nurses’ use of robots in nursing practice.

The decision to accept robots depends on nurses’ evaluations of both their advantages and disadvantages. Such evaluations would include the formation of behavioral beliefs, which comprise the attitudes toward performing the behavior [8], justifying the necessity of including behavioral beliefs and attitudes in our study.

Attitude is posited to foster behavioral intention that can further predict behavior, according to the theory of planned behavior [9]. In the nurse–robot context, planned behavior can be contextualized as nurses’ engagement with robots, defined as nurses’ actions to assist or collaborate with robots. Moreover, this theory posits that attitude is influenced by behavioral beliefs on behavioral outcomes [9]. In the nurse–robot context, behavioral beliefs can be contextualized as 2 types of beliefs among nurses: (1) those about robot benefits and (2) those about robot maintenance. Robot benefits represent improved efficiency in terms of the time and energy that robots introduce into the nursing workplace; that is, the advantages of using robots. Robot maintenance represents the effort that must be expended by nurses on robot operation; that is, the disadvantages of using robots. Robot benefits and robot maintenance thus correspond to the attitudinal antecedents in our research model. Furthermore, nurses’ personal innovativeness or the individual tendency to try new IT [10] should be important in the advanced technology context. Hence, we also included personal innovativeness as another source of encouraging nurse–robot engagement.

**Figure 1:**
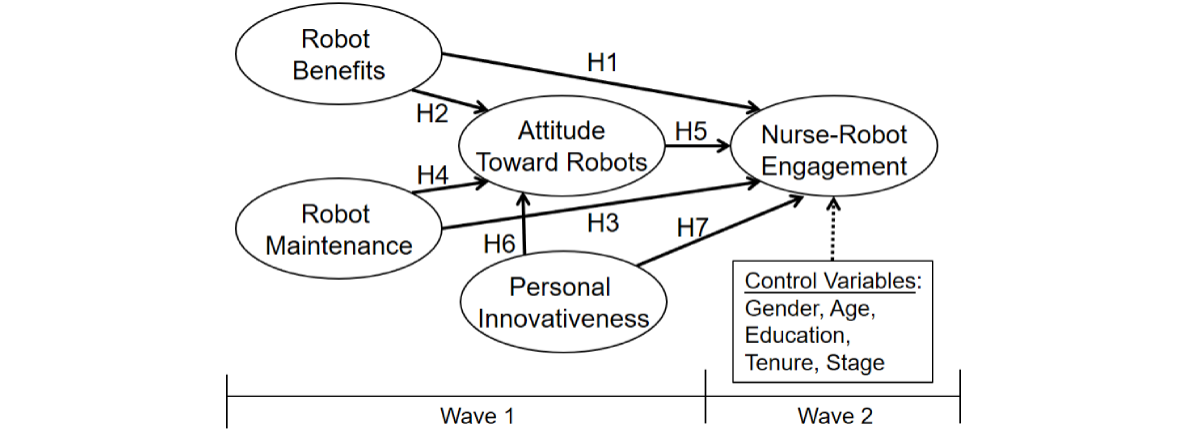
Research framework. Solid lines represent the hypothesized links. H: hypothesis.

Figure 1 illustrates the research framework, which includes concepts of robot benefits, robot maintenance, nurses’ attitudes toward robots, and nurse–robot engagement. To increase analytical rigor, we included control variables: nurses’ gender, age, education, tenure, and stage—that is, nursing skill certification [11].

We developed 7 hypotheses, indicated below as H1 to H7. Robotics is a technology advancement that brings noted efficiency and cost-saving potential [12]. We defined this as robot benefits in our study; that is, how robots help nurses optimize their time (thus alleviating their time pressure) and reduce their effort in carrying out nursing care. Robot benefits enable nurses to focus on clinical processes—supporting physicians and caring for patients—and on secondary procedures, such as carrying and moving heavy loads [13]. These benefits constitute positive reinforcements. According to reinforcement theory, positive reinforcements will promote positive behaviors [14]; for example, tending to use robots and helping ensure robots’ smooth operations. Moreover, lifting heavy loads has been frequently verified as the source of chronic lower back pain among nurses [15]. Accordingly, robots can improve nurses’ physical and psychological conditions, thus strengthening nurses’ intention to make efforts to ensure robots’ smooth operation. This behavioral intention is defined as nurse–robot engagement.

### Study Hypotheses

Our first hypothesis (H1) is that perceived robot benefits are positively related to nurse–robot engagement.

Caregivers perceive technology as useful, but they specify that it risks creating distance in their relationship with the persons in their care, and that it must complete and complement their work and not replace it [4]. That is, technologies assisting caregivers should improve caregivers’ attitudes toward such technologies. In our research context, when robots successfully assist nurses, they may create a positive attitude among nurses toward robots. The reason may be that robot assistance could be one positive environmental cue, according to reinforcement theory, which contributes to shaping desired behavior among employees [14]. Moreover, robots that can offer nurses ample time to perform their professional tasks would improve nurses’ job satisfaction and self-reported health [13]. This offers strong support that nurses will attribute their satisfaction and improved health to the assistive robots, enhancing their overall evaluation of such robots; that is, attitudes toward robots. Hence, we hypothesized that perceived robot benefits are positively related to a positive attitude toward robots (H2),

Robots can achieve outstanding performance, but also show extreme performance variation; for example, they can be out of order [16]. Such a variation may be due to insufficient fulfillment of the requirements for keeping robots operating smoothly. The required effort; that is, robot maintenance, would become a burden and a negative stimulus to frontline professionals, such as nurses. According to reinforcement theory, negative stimuli would formulate negative reinforcement and further arouse individuals’ avoidance [17,18]. In our context, such avoidance behavior is not conducive to the development of nurse–robot engagement. Moreover, workplace effort is a critical predictor of missed care [19]—an undesirable job outcome for nurses. Therefore, such maintenance effort spent on robots may make nurses reluctant to work with them. Hence, we hypothesized that perceived robot maintenance is negatively related to nurse–robot engagement (H3).

We predict that perceived robot maintenance will be negatively related to attitude toward robots. The reason is that taking extra time and effort to ensure robots’ smooth operation is not favorable to nurses. Nurses frequently need to cope with workplace time pressure [20]. Thus, any further time- and effort-related burdens (including those due to robots) would dismay them; that is, degrade their overall evaluation of the source of the burden (robots, in this case). Moreover, perceived robot maintenance implies that individuals would recognize such efforts as aversive consequences and thereby become one negative stimulus. According to reinforcement theory, negative stimuli would arouse individuals’ negative responses [17,18]; for example, an unfriendly attitude toward robots in our context. Hence, we hypothesized that perceived robot maintenance is negatively related to a positive attitude toward robots (H4).

Attitude is an overall evaluation of an object [21]. A positive attitude reflects positive feelings and expectations when approaching this object [22], motivating users’ engagement, particularly in using new technologies [23]. According to reinforcement theory, when individuals observed that pleasurable consequences would follow, they are more likely to engage in a behavior [24]. As robots have only relatively recently been introduced into nurses’ workplaces [25], we may expect that a positive attitude toward robots would also motivate nurses’ engagement. Hence, we hypothesized that a positive attitude toward robots is positively related to nurse–robot engagement (H5).

Personal innovativeness represents the individual tendency to try new IT [10]. Nurses who are digitally lagging tend to feel anxious about and avoid the use of health IT [5]. Anxiety around interacting with IT can be overcome by personal innovativeness [26], thus improving learning effectiveness in IT. Effective learning would foster correct understanding, and this sense of IT usefulness could promote nurses’ positive attitudes toward robot use [27]. Personal innovativeness encourages nurses to obtain IT knowledge [28], thus enhancing their positive evaluations of working with IT. As with all other IT, robots require user knowledge to allow meaningful collaboration. Hence, personal innovation and its effect on obtaining robot knowledge are also important in determining positive evaluations of working with robots; that is, attitudes toward robots. Furthermore, personal innovativeness enhances positive attitudes toward a new technology—for example, mobile payment [29]—thus also supporting this link. Accordingly, we hypothesized that nurses’ personal innovativeness is positively related to a positive attitude toward robots (H6).

Personal innovativeness is an important aspect of technology use; for example, smartphone use [30]. We argue that this also applies to robot use. The reason is that personal innovativeness is the general tendency to try technology applications [10]. Applied to our research context, high levels of personal innovativeness would encourage robot use among nurses—for example, trying robots and exploring their functions—thus better understanding the use of and using (or engaging with) robots in health care workplaces. Hence, we hypothesized that nurses’ personal innovativeness is positively related to nurse–robot engagement (H7).

After the development of the study hypotheses, we describe the methods to test them.

## Methods

### Sample and Data Collection Process

Our study used a 2-wave follow-up design. We surveyed registered nurses in operating rooms at a large-scale medical center in Taiwan. The first wave was conducted from October to November 2019, while the participants were followed up in the second wave, which was conducted from December 2019 to February 2020. We chose nurses working in operating rooms as the study participants because robots were assistive in those operating rooms, thus meeting our research purpose. Assistive robots are shown in Multimedia Appendix 1. They help carry heavy equipment and materials that are necessary for surgical procedures. One assistive robot completed more than 200 trips per day (average distance 200 m per trip) between the storage and the 60 operating rooms in an area larger than 9000 m^2^. That is, such assistive robots greatly reduced the time for preparing those equipment and materials and boosted nurses’ time for direct patient care in operating rooms.

We used a census method to maximize sample representativeness because it avoids any sampling error. By definition, a census method includes all eligible participants; thus, estimating any sample size is not appropriate. Nonetheless, we calculated the estimated sample size to ensure that the testing power is sufficient. Specifically, we found that totally 358 nurses met our eligibility criteria. We then consulted Raosoft [31] to estimate the required sample size. We used the typical standard—that is, 95% confidence level and a 50% response distribution—for computations. We finally obtained an estimated sample size of 186 individuals—that is, we would need to include at least 186 participants in our study.

We included full-time registered nurses. Based on our exclusion criteria, nursing students, nursing practitioners, nurse interns, and nursing supervisors were excluded. In total, the 358 eligible nurses in the operating rooms were approached. We obtained the consent of 344 nurses and used their responses in the first wave. We were unable to follow up with 13 nurses in the second wave. Hence, 331 nurses returned completed responses and were included in our formal analyses.

### Ethical Considerations

This study was approved by the institutional review board of Chang Gung Memorial Hospital (approval 201900311B0C602). All included nurses were informed of the study objective, and written consent was sought for their participation in the study. All participants were volunteers and could quit the study at any time without giving a reason. Completed responses were collected by research assistants who did not work at the medical center, thus keeping responses confidential and ensuring voluntary participation.

### Measurements

Items measuring robot benefits and robot maintenance were developed on the basis of those of Kohli et al [32] and the senior nurses who worked with robots in our context. We modified the items of You and Robert [33] to measure nurses’ attitudes toward robots. We adapted the items of Chang et al [34] to measure nurse–robot engagement. Items measuring personal innovativeness were obtained from Kalinic and Marinkovic [35]. All the items are listed in Multimedia Appendix 2. For all items, we used a 5-point Likert scale with 1=“strongly disagree” and 5=“strongly agree.” High scores represent high levels of the measured constructs. As we have adapted the study items, we needed to evaluate the reliability and validity of our measures.

### Data Analysis

To implement the structural equation modeling technique to test our hypotheses, we used LISREL (linear structural relations; version 8.80; Scientific Software International). For all other analyses, we used SPSS (version 17.0; IBM Corp).

Our dependent variable was nurse–robot engagement. Our independent variables were as follows: robot benefits, robot maintenance, nurses’ attitudes toward robots, and personal innovativeness. We included 5 control variables: (1) nurses’ gender, (2) nurses’ age, (3) nurses’ education level, (4) tenure (ie, number of years working as a nurse), and (5) stage (ie, nursing skill as evaluated from accreditation levels N1 to N4 according to Teng et al [11]. We obtained 0.4% of missing values, which is negligible. Thus, we used complete case analyses. We set a typical significance level at .05.

## Results

### Psychometric Properties

Table 2 lists the loadings and cross-loadings. All the items loaded on the assumed factors. We did not observe significant cross-loadings, which preliminarily supports our data validity.

Table 3 lists the Pearson correlations and psychometric properties of our study constructs. The minimum correlation was –0.11, while the maximum correlation was 0.66. These correlations were not high (<0.70), not indicating common method variance (CMV). We also used the method of Podsakoff et al [36] to examine CMV. We found that CMV was unlikely to interfere with our model (Δ*χ*^2^_40_=7661.9>*χ*^2^_40_=55.8; α=.05). Moreover, our study used a 2-wave follow-up design, offering temporal separation between constructs and thus also negating CMV [36]. Moreover, the psychometric properties listed in Table 3 are acceptable.

As listed in Table 3, items measuring each construct had a Cronbach α of ≥.90, composite reliability of ≥0.90, and average variance extracted of ≥0.69, which together suggest adequate reliability. All our items had loadings of >0.80 (Multimedia Appendix 2), which indicates convergent validity. All the positive square roots of average variance extracted values were larger than the counterpart correlation coefficients, demonstrating sufficient discriminant validity. The measurement model fit the data acceptably criteria (ie, comparative fit index=0.96, incremental fit index=0.96, nonnormed fit index=0.95, and root mean square error of approximation=0.076).

**Table 2:** Loadings and cross-loadings.

	Robot benefit, indicator loading (λ)	Robot maintenance, λ	Attitude toward robots, λ	Personal innovativeness, λ	Nurse–robot engagement, λ
Robot benefits-1	*0.82* ^a^	–.03	0.12	0.08	0.19
Robot benefits-2	*0.89*	–.02	0.17	0.10	0.22
Robot benefits-3	*0.92*	–.00	0.21	0.11	0.16
Robot benefits-4	*0.89*	–.07	0.15	0.09	0.20
Robot benefits-5	*0.93*	–.02	0.18	0.11	0.11
Robot benefits-6	*0.91*	–.02	0.19	0.11	0.18
Robot maintenance-1	0.00	*0.90*	–0.03	0.01	–0.02
Robot maintenance-2	–0.04	*0.96*	–0.03	0.05	–0.00
Robot maintenance-3	–0.04	*0.97*	–0.04	0.06	0.01
Robot maintenance-4	–0.03	*0.97*	–0.04	0.06	–0.01
Robot maintenance-5	–0.02	*0.97*	–0.05	0.06	–0.01
Robot maintenance-6	–0.03	*0.97*	–0.04	0.04	0.00
Attitude toward robots-1	0.41	–0.01	0.27	0.15	*0.80*
Attitude toward robots-2	0.42	0.01	0.29	0.15	*0.80*
Attitude toward robots-3	0.45	–0.01	0.24	0.13	*0.73*
Personal innovativeness-1	0.10	–0.02	0.09	*0.88*	0.20
Personal innovativeness-2	0.11	0.00	0.10	*0.86*	0.21
Personal innovativeness-3	0.09	0.11	0.08	*0.88*	0.00
Personal innovativeness-4	0.15	0.15	0.13	*0.82*	–0.06
Nurse–robot engagement-1	0.22	–0.05	*0.90*	0.10	0.13
Nurse–robot engagement-2	0.23	–0.07	*0.90*	0.09	0.17
Nurse–robot engagement-3	0.18	–0.06	*0.90*	0.13	0.17
Nurse–robot engagement-4	0.21	–0.06	*0.89*	0.13	0.15

^a^Italicized values are indicator loading (λ) values in the theoretically assumed factors.

**Table 3:** Correlations among the study constructs.

	Robot benefits	Robot maintenance	Attitude toward robots	Personal innovativeness	Nurse–robot engagement
Robot benefits	1.00				
Robot maintenance	–0.06	1.00			
Attitude toward robots	0.66^a^	–0.03	1.00		
Personal innovativeness	0.27^a^	0.11	0.32^a^	1.00	
Nurse–robot engagement	0.43^a^	–0.11	0.52^a^	0.26^a^	1.00
Mean (SD)	3.89 (0.86)	3.06 (1.03)	3.99 (0.79)	3.65 (0.75)	4.02 (0.77)
Cronbach α	.97	.98	.93	.90	.96
Composite reliability	0.97	0.97	0.93	0.90	0.95
Average variance extracted	0.83	0.85	0.82	0.69	0.82

^a^Significant at *P*<.05.

### Participant Profiles

Table 4 depicts our participant profile: 311 (94.0%) were female, 307 (92.8%) were aged between 20 and 50 years, 250 (75.5%) attended college or university, 217 (65.6%) ranked N3 or N4, and 233 (70.4%) worked as a nurse for 5 or more years. The gender composition resembles the composition of the nursing body in Taiwan (96.7%) [37].

**Table 4:** Profile of the participants (N=331).

Variables		Nurses, n (%)
**Gender**		
	Female	311 (94.0)
	Male	19 (5.7)
	Missing	1 (0.3)
**Age (years)**		
	20-30	122 (36.9)
	30-40	78 (23.6)
	40-50	107 (32.3)
	50-60	23 (6.9)
	≥60	1 (0.3)
**Education level**		
	High school or lower	80 (24.2)
	College or university	241 (72.8)
	Graduate institute	9 (2.7)
	Missing	1 (0.3)
**Stage**		
	N1 (basic nursing)	57 (17.2)
	N2 (acute nursing)	56 (16.9)
	N3 (holistic nursing)	95 (28.7)
	N4 (specialty nursing)	122 (36.9)
	Missing	1 (0.3)
**Tenure (years)**		
	<1	13 (3.9)
	≥1 and <5	61 (18.4)
	≥5 and <10	50 (15.1)
	≥10 and <15	33 (10.0)
	≥15 and <20	51 (15.4)
	≥20	99 (29.9)
	Missing	24 (7.3)

### Hypothesis Testing

**Figure 2:**
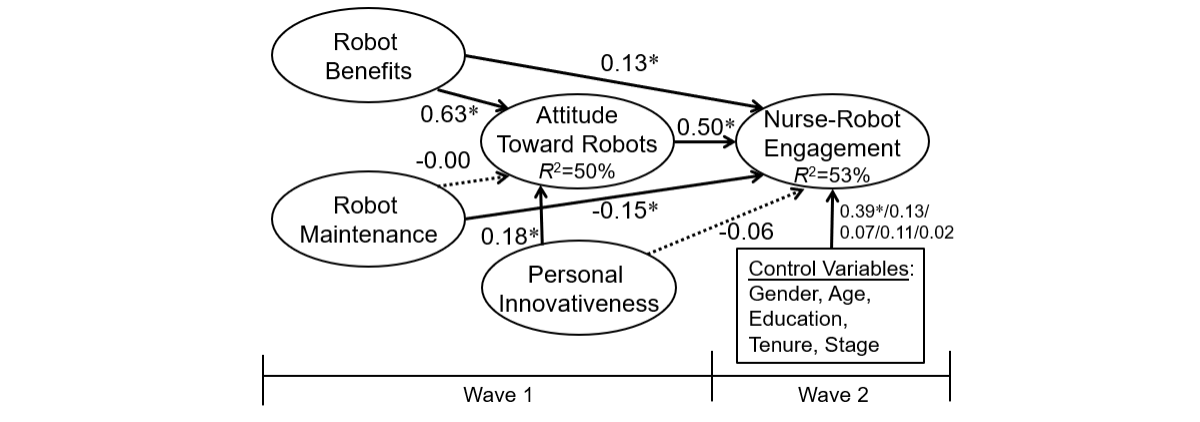
Analytical results. *P<.05.

Figure 2 illustrates the testing results. All but 2 hypotheses were supported. The first exception is the insignificant relation between robot maintenance and nurses’ attitudes toward robots (*β*=–.00; *P*=.92), not supporting H4. The second exception is the insignificant relation between personal innovativeness and nurse–robot engagement (*β*=–.06; *P*=.27), not supporting H7.

Our structural model fit the data acceptably (CFI=0.96, IFI=0.96, NNFI=0.95, RMSEA=0.075). The RMSEA is acceptable [38]. Moreover, not all indices are expected to perform perfectly [39]. Our model explained a large proportion of the variance in the dependent variable; that is, 53% of nurse–robot engagement, representing moderately large effect sizes [40].

## Discussion

### Principal Findings and Contributions

Our study is the first to examine how robot benefits and robot maintenance of assistive robots result in nurse–robot engagement. The findings uniquely indicate that the robot benefits are an important source of nurses’ engagement with robots, while nurses’ attitudes toward robots is important in motivating their engagement with robots. Overall, our study contributes to the literature on artificial intelligence–based technologies in the health care context; that is, by illustrating the relative importance of robot benefits, when compared to robot maintenance.

The reason for nonsupported H4 may be that the hospital had its own robot maintenance team. Thus, nurses would not be required to perform this work, thus creating no barrier to the acceptance of robots and not substantially harming the attitudes toward robots. The reason for nonsupported H7 may be that helpful robots should be widely accepted by nurses, reducing the difference generated by personal innovativeness.

Overall, this study makes several contributions. First, it examines the means (eg, increasing robot benefits) to increase nurses’ engagement with robots. Second, it points out that robot benefits are highly important. Third, it identifies that nurses’ attitudes toward robots play a pivotal role by transforming personal innovativeness into enhanced engagement; that is, instilling a positive attitude among health care professionals toward robots is critical for boosting their engagement with them.

### Theoretical Implications

This study reports novel means (eg, increasing robot benefits) to increase health care professionals’ engagement with artificial intelligence–based technologies, particularly in a context in which assistive robots were used by nurses.

Boumans et al [41] verified the benefits of an assistive social robot. Our study is in line with theirs in examining the benefits of assistive robots in health care contexts. However, our study examined new benefits; that is, saving labor by carrying heavy equipment and materials. Such benefits could positively contribute to nurses’ health [13], which improves their performance [41].

De Leeuw et al [5] examined the antecedents that influence nurses’ digital lagging in their adoption of health information technology. Our study is in concordance with theirs in investigating nurses’ attitudes toward health information technology. Uniquely, our findings indicate that 2 factors—enhanced robot benefits and reduced robot maintenance—can increase nurses’ engagement with robots and demonstrate their relative importance in attitude formation. Chang et al [13] examined the pros and cons of using assistive robots on task engagement. Our study is new in examining *robot engagement*—that is, how much nurses use robots—but not *task engagement*—that is, how much time nurses spend in delivering professional care. Moreover, all the constructs in our study were not included in Chang et al’s [13] study, further exhibiting the novelty of our study.

The interesting findings of our study offer several unique theoretical contributions. First, we find that, compared to negative IT characteristics (robot maintenance) and user characteristics (personal innovativeness), only positive IT characteristics (robot benefits) can directly influence both nurses’ technology evaluation (attitudes toward robots) and technology use (nurse–robot engagement). Second, another interesting finding is that IT characteristics (robot benefits and maintenance) have a much stronger influence than user characteristics (personal innovativeness) on improving technology use. Third, user characteristics (personal innovativeness) are more important than negative IT characteristics (robot maintenance) in improving technology evaluation. The unbalanced role of negative IT characteristics in decreasing technology use while sustaining technology evaluation presents an interesting finding, which deserves more research, thus demonstrating the fourth contribution.

### Implications in Practice

Our study was implemented in a large medical center, in which assistive robots were used in a number of operating rooms. Moreover, this medical center had its own IT maintenance team for robot maintenance. Our findings may be generalized to international contexts that share similar features with this medical center.

We found that robot benefits can enhance nurse–robot engagement. Specifically, we measured robot benefits by harnessing 2 characteristics: time saving and energy keeping. Hence, it is suggested that health care robot developers should focus on designing their robots to save more time and energy for users. The robot benefits should be promoted when communicating with hospital management responsible for robot introduction. Moreover, hospital management could in turn consider practical training to introduce the robots into the work space, familiarize nurses with their functioning, and reiterate their benefits to nurses.

Our findings also envision hospital management that are eager to introduce artificial intelligence–based technologies into their hospitals. Intuitively, health care professionals are not experts on artificial intelligence–based technologies and many may not be ready to fully engage with them. Training could be offered to nurses to do easy robot maintenance and show them evidence regarding robot benefits, thus increasing nurse–robot engagement. This finding paves the way to increasing this engagement; that is, informing health care professionals of robot benefits that help them serve better.

We also found that personal innovativeness helps improve health care professionals’ attitudes toward robots, thereby increasing their engagement with the robots. This finding suggests that hospital management could evaluate the personal innovativeness of health care professionals; for example, through a short survey, and empowers those who are willing to try new technologies as project champions to promote robot use within their work units. As these project champions are keen to interact with new technologies, this will best spread the use of intelligent technologies within the hospitals.

### Limitations and Future Research Directions

Our study was implemented in a large-scale medical center in Taiwan. Future studies could replicate ours at multiple research sites and in multiple countries, incorporating more organizational or cultural factors to deepen our understanding of intelligent technology use.

Our study adopts a 2-wave design. This design offers evidence supporting the temporal sequence of the causal influences. Future studies could adopt qualitative designs to understand in depth the mechanisms underlying our study findings.

This study was implemented in operating rooms. Hence, we were restrained from knowing whether the findings are generalizable to patient wards or outpatient departments. This study did not directly include robot features and therefore restrained us from knowing the influence of each robot feature. Future studies could seek means to address the limitations of this study.

### Conclusions

Our study examined how health care professionals—that is, nurses—evaluated intelligent technologies, in a context where nurses evaluate assistive robots. Our findings uniquely indicate the importance of robot benefits as a key driver to form positive attitudes toward robots and to increase nurse–robot engagement. We obtained an interesting finding that robot benefits have a stronger influence on nurse–robot engagement than robot maintenance needs. This finding envisions the developers of artificial intelligence–based technology—for example, robot developers—focusing on delivering and communicating the best robot functions to health care professionals while simultaneously focusing on reducing maintenance as a secondary issue.

## Appendix

Multimedia Appendix 1 Assistive robots in this study.

Multimedia Appendix 2 Measurement items.

## Abbreviations

CMV: common method variance
LISREL: linear structural relations
