# Supplementary material for: Enhancing Nurse–Robot Engagement: Two-Wave Survey Study
Source: J Med Internet Res. 2023 Jan 9;25:e37731. doi: 10.2196/37731 (PMC9893885; doi:10.2196/37731)
Supplement: Multimedia Appendix 1 [file jmir_v25i1e37731_app1.docx]

**Multimedia Appendix 1: Assistive Robots in This Study**

| 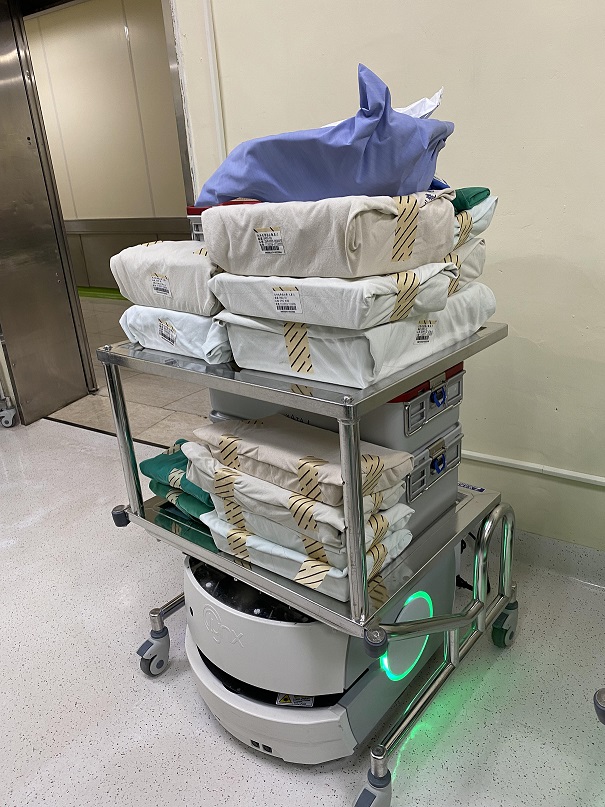 | 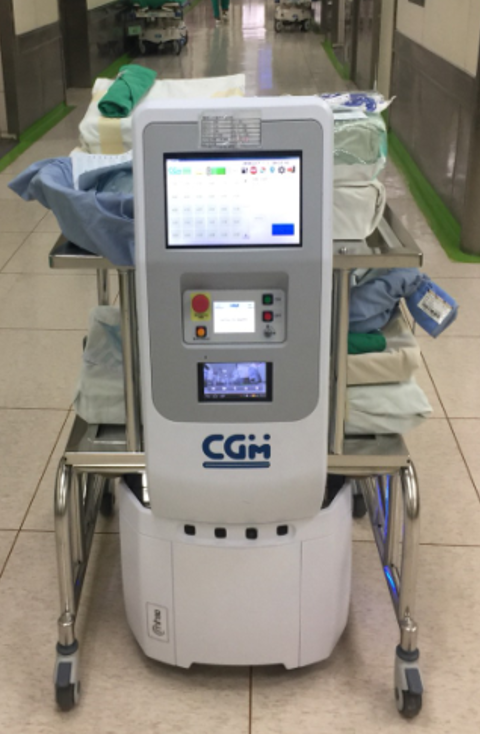 |
| --- | --- |
| **Figure A1: Assistive Robot Reaching Operating Room** | **Figure A2: Control Panel of Assistive Robot** |
