# Supplementary material for: Enhancing Nurse–Robot Engagement: Two-Wave Survey Study
Source: J Med Internet Res. 2023 Jan 9;25:e37731. doi: 10.2196/37731 (PMC9893885; doi:10.2196/37731)
Supplement: Multimedia Appendix 2 [file jmir_v25i1e37731_app2.docx]

**Multimedia Appendix 1: Measurement Items**

| Construct | Item | *M* | *SD* | *λ* |
| --- | --- | --- | --- | --- |
| **Robot Benefits** | Robots save my time by carrying equipment and medical materials. | 4.04 | 0.90 | .83 |
|  | As the equipment and medical materials are carried by robots, I have more time to take care of patients. | 3.85 | 0.96 | .91 |
|  | As the equipment and medical materials are carried by robots, I have more time to allocate and use. | 3.86 | 0.92 | .94 |
|  | Robots save me effort by carrying equipment and medical materials. | 3.93 | 0.90 | .91 |
|  | As the equipment and medical materials are carried by robots, I have more energy to take care of patients. | 3.81 | 0.95 | .93 |
|  | As the equipment and medical materials are carried by robots, I have more energy to allocate and use. | 3.84 | 0.91 | .94 |
| **Robot Maintenance** | I need to spend extra time to maintain the robots’ continuous operation. | 3.19 | 1.09 | .84 |
|  | I need to spend extra time to make the robots work effectively. | 3.06 | 1.07 | .91 |
|  | I need to spend extra time to ensure that the robots can maintain their efficient operation. | 3.03 | 1.08 | .92 |
|  | I need to expend extra effort to maintain the robots’ continuous operation. | 3.02 | 1.07 | .95 |
|  | I need to expend extra effort to make the robots work effectively. | 3.03 | 1.07 | .95 |
|  | I need to expend extra effort to ensure that the robots can maintain their efficient operation. | 3.01 | 1.08 | .94 |
| **Attitude toward Robots** | I would feel comfortable if I were given a job where I had to use robots. | 3.90 | 0.88 | .94 |
|  | I would feel comfortable when operating a robot in front of my peers. | 3.96 | 0.85 | .96 |
|  | I think it’s a good thing that robots can operate autonomously. | 4.13 | 0.78 | .82 |
| **Personal Innovativeness** | I am eager to learn about new technologies. | 3.93 | 0.82 | .82 |
|  | I am eager to try new technologies. | 3.95 | 0.80 | .81 |
|  | I think I am one of the first among my peers to have tried new technologies. | 3.39 | 0.89 | .88 |
|  | My peers often come to me for advice about new technologies and innovation. | 3.31 | 0.90 | .81 |
| **Nurse–Robot Engagement** | I am willing to give some assistance to robots. | 3.98 | 0.82 | .93 |
|  | I am willing to help robots to ensure their continuous running. | 4.04 | 0.83 | .94 |
|  | I tend to assist robots when they encounter difficulties. | 4.05 | 0.82 | .88 |
|  | When robots encounter a difficult situation, I will help them and ensure they operate smoothly. | 3.99 | 0.82 | .87 |

*Note*. *M*=average; *SD*=standard deviation; *λ*=indicator loading.
